# Late Renal Cell Carcinoma Metastasis Mimicking an Arteriovenous Malformation in the Parotid and Masseter Region: A Diagnostic Pitfall

**DOI:** 10.7759/cureus.110487

**Published:** 2026-06-08

**Authors:** Luis A Huerta Diaz, Brandon A Lopez Alanis, Flor E Ortiz Villeda, Marco A Treviño Lozano, Gerardo E Muñoz-Maldonado

**Affiliations:** 1 General Surgery, Hospital Universitario “Dr. José E. González” de la Universidad Autónoma de Nuevo León (UANL), Monterrey, MEX; 2 Pathology, Hospital Universitario “Dr. José E. González” de la Universidad Autónoma de Nuevo León (UANL), Monterrey, MEX

**Keywords:** arteriovenous malformation, clear cell rcc, hypervascular mass, late metastasis, masseter muscle, parotid metastasis, renal cell carcinoma

## Abstract

Renal cell carcinoma (RCC) is characterized by unpredictable metastatic behavior and the potential for recurrence many years after initial treatment. Metastases to the head and neck region are uncommon, and involvement of the masseter muscle is exceptionally rare, often creating a diagnostic challenge. We report the case of a 77-year-old man with a history of radical nephrectomy for RCC 12 years earlier who presented with a painless, progressively enlarging mass in the right parotid region. Imaging studies demonstrated a markedly hypervascular lesion with high-flow characteristics, initially suggestive of an arteriovenous malformation. However, the patient’s oncologic history prompted further investigation, and ultrasound-guided fine-needle aspiration confirmed metastatic clear cell RCC. He underwent superficial parotidectomy with en bloc resection of the masseteric lesion. Histopathologic examination and immunohistochemical staining demonstrating focal PAX8 positivity and diffuse carbonic anhydrase IX (CAIX) expression confirmed the diagnosis. This case highlights the ability of late RCC metastases to mimic vascular lesions because of their marked hypervascularity, particularly in unusual anatomical locations. Metastatic RCC should remain in the differential diagnosis of hypervascular head and neck masses, even after prolonged disease-free intervals.

## Introduction

Renal cell carcinoma (RCC) is characterized by its unpredictable metastatic behavior and well-recognized potential for late recurrence. Metastatic disease may develop many years after apparently curative nephrectomy, even in patients who have remained disease-free during prolonged follow-up, posing an ongoing diagnostic challenge for clinicians [1].

Although the lungs, bone, and liver are the most common sites of dissemination, RCC can occasionally involve the head and neck region. Among these uncommon presentations, metastases to the parotid gland are rare and account for only a small proportion of salivary gland malignancies [2-4]. Skeletal muscle involvement is even less frequent despite the rich vascular supply of these tissues. Within this group, metastasis to the masseter muscle is exceptionally uncommon and has been described only in isolated reports [5-7].

Clear cell RCC is a highly vascular neoplasm that frequently demonstrates intense contrast enhancement and prominent vascularity on imaging studies. Consequently, metastatic lesions may mimic vascular abnormalities, creating diagnostic uncertainty and potentially delaying appropriate management [8]. This challenge is particularly relevant in the head and neck region, where hypervascular lesions encompass a broad differential diagnosis.

We report a rare case of delayed RCC metastasis involving the parotid-masseteric region that presented 12 years after nephrectomy and was initially interpreted as an arteriovenous malformation because of its marked hypervascular appearance. The case highlights the importance of correlating imaging findings with oncologic history and maintaining suspicion for metastatic RCC when evaluating hypervascular head and neck masses, even after prolonged disease-free intervals.

## Case presentation

A 77-year-old man presented with a one-year history of a progressively enlarging mass in the right parotid region. His medical history was notable for systemic hypertension, hypothyroidism treated with levothyroxine 100 μg daily, and a left radical nephrectomy with splenectomy performed 12 years earlier for renal cell carcinoma. No evidence of recurrence had been documented during follow-up.

On presentation, the patient was hemodynamically stable, with a blood pressure of 135/86 mmHg, heart rate of 74 beats/minute, respiratory rate of 18 breaths/minute, temperature of 36.7°C, and oxygen saturation of 98% on room air. He denied pain, dysphagia, facial weakness, weight loss, or other constitutional symptoms. Physical examination revealed a well-circumscribed, firm, mobile mass measuring approximately 3 cm in the right parotid region without overlying skin changes. No cervical lymphadenopathy was identified, and facial nerve function was intact.

**Figure 1:**
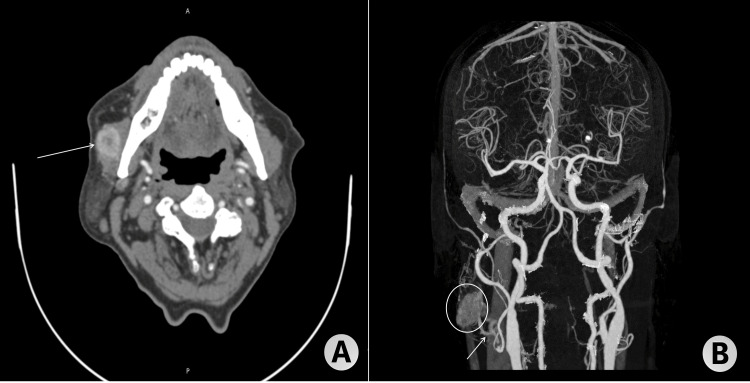
Imaging findings of the right parotid-masseteric lesion (A) Axial CECT demonstrating a well-defined, hyperenhancing mass in the right parotid-masseteric region (arrow), consistent with a hypervascular lesion. (B) Three-dimensional CT angiographic reconstruction showing a highly vascular mass (circle) with prominent arterial supply from branches of the external carotid system (arrow), initially suggestive of a high-flow vascular malformation. CECT: contrast-enhanced CT

Contrast-enhanced CT (CECT) demonstrated a well-defined hypervascular lesion centered within the right masseter muscle. Axial images showed intense contrast enhancement in the parotid-masseteric region (Figure 1A). Three-dimensional CT angiographic reconstruction demonstrated marked vascularity with prominent arterial feeders, raising concern for a high-flow vascular malformation (Figure 1B). 

Given the patient's history of RCC and the presence of a discrete solid lesion, ultrasound-guided fine-needle aspiration was performed. Cytologic evaluation was reported as consistent with metastatic clear cell RCC and contributed to preoperative planning; however, original cytology images were not available for review. Additional staging studies performed before surgery revealed no evidence of metastatic disease elsewhere.

**Figure 2:**
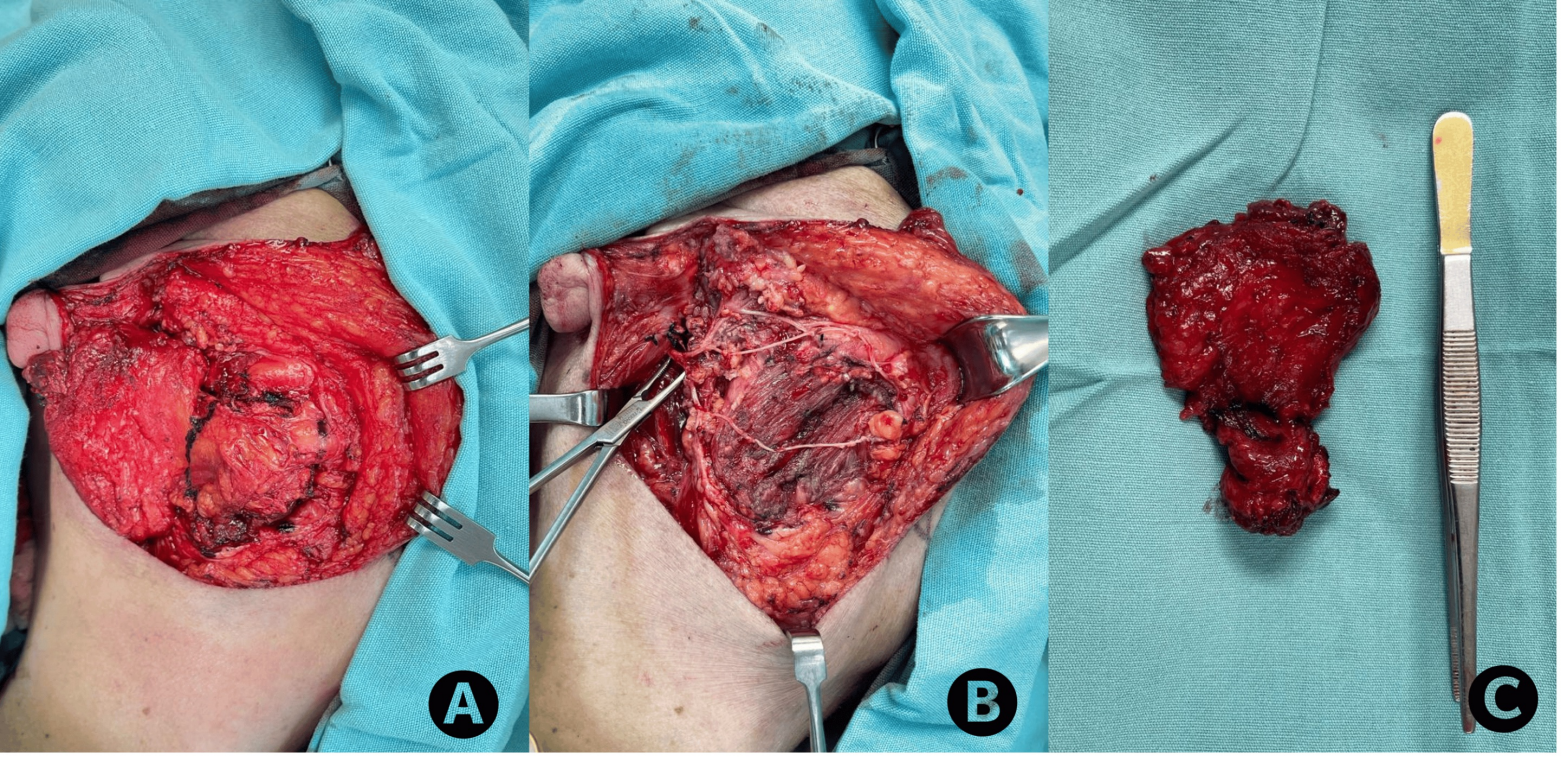
Intraoperative findings and surgical specimen (A) Initial surgical exposure demonstrating a hypervascular lesion within the right parotid-masseteric region. (B) Deep dissection showing involvement of the masseter muscle during lesion mobilization and resection. (C) Gross surgical specimen following en bloc resection.

The patient underwent superficial parotidectomy with en bloc resection of the masseteric lesion. Intraoperatively, a well-demarcated but highly vascular lesion involving the masseter muscle was identified. Careful dissection allowed identification and preservation of the facial nerve branches. The operative field during initial exposure and deep dissection is shown in Figure 2A and Figure 2B, respectively. The resected specimen is shown in Figure 2C.

**Figure 3:**
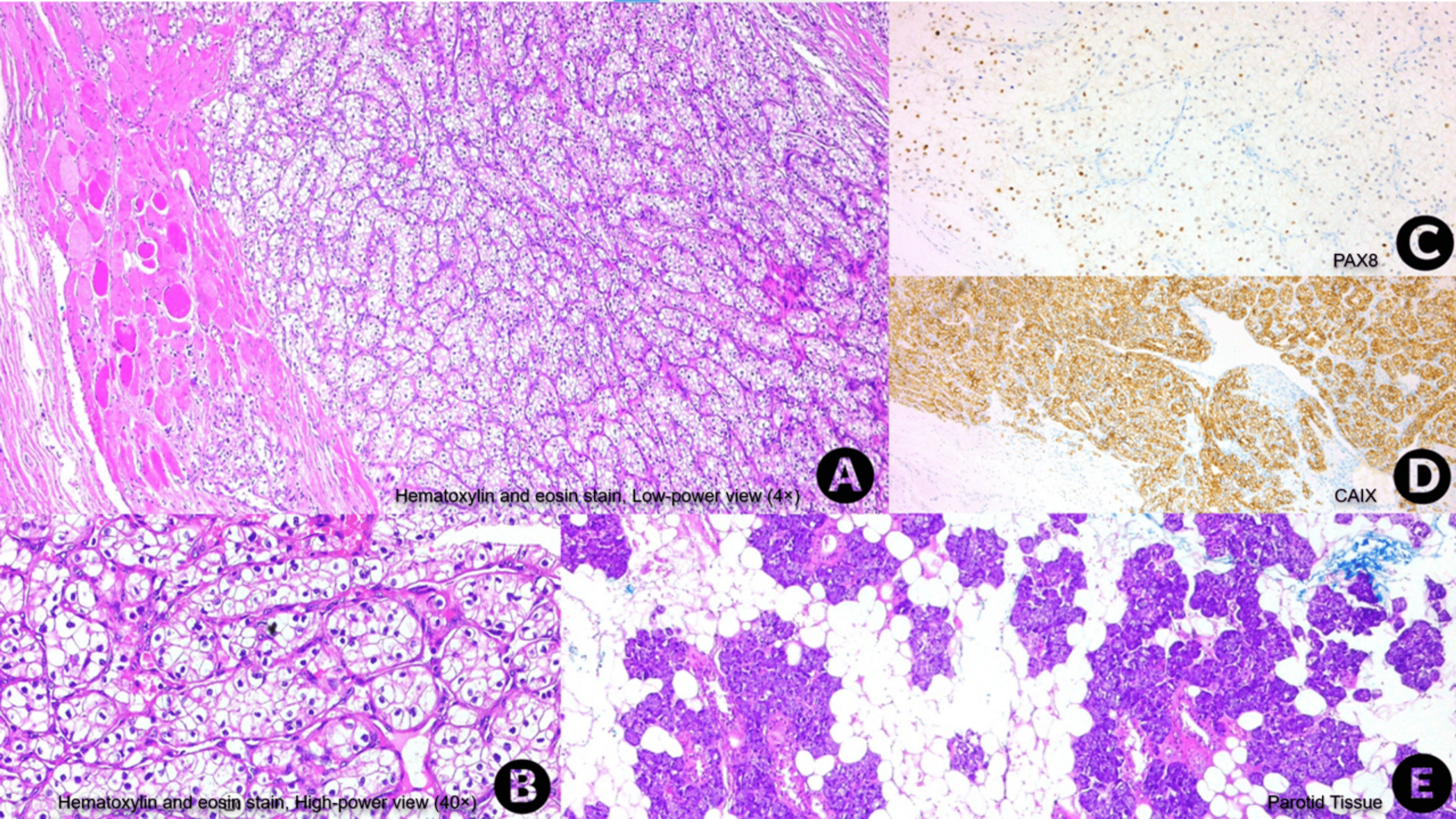
Histopathological and immunohistochemical findings (A) Hematoxylin and eosin stain, Low-power view (4×) demonstrating a nodular proliferation of neoplastic cells infiltrating skeletal muscle fibers. (B) Hematoxylin and eosin stain, High-power view (40×) showing tumor cells arranged in nests separated by delicate vascular septa, with prominent nuclei and clear cytoplasm. (C) Immunohistochemistry demonstrating focal nuclear positivity for PAX8. (D) Strong and diffuse cytoplasmic staining for carbonic anhydrase IX (CAIX). (E) Adjacent parotid tissue without significant histopathological alterations.

Histopathological examination confirmed metastatic clear cell RCC involving skeletal muscle. The tumor demonstrated a nodular proliferation of clear cells with a rich vascular network infiltrating muscle fibers (Figures 3A, 3B). Immunohistochemical analysis showed focal nuclear positivity for PAX8 (Figure 3C) and strong diffuse expression of carbonic anhydrase IX (CAIX) (Figure 3D), supporting renal origin. Adjacent parotid tissue showed no significant histopathological alterations (Figure 3E). Surgical margins were free of tumor.

The postoperative course was uneventful, with preservation of facial nerve function. The patient was discharged in stable condition and referred for oncologic follow-up. At the six-month follow-up, no evidence of local recurrence was identified.

## Discussion

The present case illustrates two characteristic features of RCC: the potential for late recurrence and the ability to metastasize to uncommon anatomical locations. Although metastatic involvement of the head and neck region is uncommon, the parotid gland has been recognized as a potential site of dissemination [2-4]. Skeletal muscle metastases are considerably less frequent, and involvement of the masseter muscle remains exceptionally rare, with only isolated cases reported in the literature [5-7].

Previously reported cases of RCC metastasis involving the parotid gland or masseter muscle are summarized in Table 1. Compared with prior reports, the present case is notable for the combination of a prolonged disease-free interval, simultaneous involvement of the parotid-masseteric region, and an imaging appearance initially suggestive of a high-flow vascular malformation.

**Table 1:** Selected reported cases of renal cell carcinoma metastasis involving the parotid gland or masseter muscle RCC: renal cell carcinoma; AVM: arteriovenous malformation.

Author (Year)	Site of metastasis	Disease-free interval	Relevant imaging/pathologic findings	Treatment
Spreafico et al. (2008) [2]	Parotid gland with masseter involvement	Delayed recurrence after nephrectomy	Parotid-region metastatic RCC	Parotidectomy
Park and Hlivko (2002) [3]	Parotid gland	6 years	Hypervascular parotid mass	Parotidectomy
Mrena et al. (2008) [4]	Parotid gland	Delayed recurrence	Histopathologic review of parotid metastases	Surgical management
Gal et al. (1997) [5]	Masseteric space	Not specified	Clear cell neoplasm within masticator space	Surgical excision
Bae et al. (2012) [6]	Masseter muscle	Synchronous presentation	Hypervascular intramasseteric lesion	Surgery
Qin et al. (2022) [7]	Masseter muscle	5 years	Hypervascular masseter metastasis from RCC	Complete metastasectomy
Present case (2026)	Parotid-masseteric region	12 years	Hypervascular lesion mimicking AVM	Superficial parotidectomy with en bloc resection

One of the most clinically relevant aspects of this case is the 12-year interval between nephrectomy and metastatic presentation. Late recurrence is a well-recognized characteristic of RCC and may occur many years after apparently curative treatment [1]. This prolonged latency may reduce clinical suspicion and delay diagnosis, particularly when lesions arise in atypical locations and present with nonspecific symptoms.

The radiologic appearance of the lesion represented an additional diagnostic challenge. Clear cell RCC is characteristically hypervascular and frequently demonstrates intense contrast enhancement and prominent vascularity on imaging studies. As a result, metastatic deposits may mimic vascular abnormalities, including arteriovenous malformations, creating potential diagnostic confusion [8]. In the present case, CECT and angiographic reconstruction demonstrated marked vascularity, initially favoring the diagnosis of a high-flow vascular lesion. However, the patient's oncologic history and the presence of a discrete solid mass prompted tissue sampling before definitive treatment.

Histopathological examination and immunohistochemistry were essential for establishing the diagnosis. The characteristic morphology of clear cell RCC, including nests of clear cells separated by delicate vascular septa, was supported by immunohistochemical staining. PAX8 positivity is a useful marker of renal origin, while strong expression of CAIX is highly characteristic of clear cell RCC and may assist in distinguishing metastatic RCC from primary salivary gland neoplasms with clear cell features [9,10].

Complete surgical resection remains an appropriate treatment strategy for isolated and technically resectable metastases. In the present case, superficial parotidectomy with en bloc resection of the masseteric lesion achieved complete excision with negative surgical margins while preserving facial nerve function. No local recurrence was identified during six months of follow-up.

This case highlights the importance of maintaining suspicion for metastatic RCC when evaluating hypervascular head and neck masses, even after prolonged disease-free intervals. Correlation of imaging findings with oncologic history and histopathologic confirmation remains essential to avoid misdiagnosis and to guide appropriate management.

## Conclusions

Metastatic RCC should remain in the differential diagnosis of hypervascular head and neck masses, even after prolonged disease-free intervals following nephrectomy. This case illustrates the potential for delayed RCC metastasis to involve uncommon sites such as the parotid-masseteric region and to radiologically mimic a high-flow vascular malformation. Correlation of imaging findings with oncologic history and histopathologic confirmation is essential for accurate diagnosis and appropriate management. Complete surgical resection can provide effective local control in selected patients with isolated metastatic disease.
